# Supplementary material for: Cell Free Methylated Tumor DNA in Bronchial Lavage as an Additional Tool for Diagnosing Lung Cancer—A Systematic Review
Source: Cancers (Basel). 2022 Apr 30;14(9):2254. doi: 10.3390/cancers14092254 (PMC9099950; doi:10.3390/cancers14092254)
Supplement: Supplementary file 1 [file cancers-14-02254-s001.zip › cancers-1684273-supplementary.pdf]

cancers

MDPI

Review

Cell free methylated tumor DNA in bronchial lavage as an additional tool for diagnosing lung cancer – A systematic review

Sara Witting Christensen Wen[1,2*], Jan Wen[3], Torben Frøstrup Hansen[1,2], Anders Jakobsen[1,2] and Ole Hilberg[2,4]

Supplementary Materials

1. Detailed database searches

PubMed: Searched on February 25, 2022.

Block 1:
(bronchiolo-alveolar adenocarcinomas) OR (bronchiolo-alveolar adenocarcinoma) OR (bronchiolo alveolar adenocarcinomas) OR (bronchiolo alveolar adenocarcinoma) OR (carcinoma, bronchogenic) OR (bronchial neoplasms) OR (bronchial neoplasm) OR (bronchial carcinomas) OR (bronchial carcinoma) OR (squamous cell lung cancer) OR (lung squamous cell carcinoma) OR (squamous cell carcinoma lung) OR (lung adenocarcinoma) OR (adenocarcinoma lung) OR (small cell lung carcinoma) OR (small cell lung cancer) OR (non small cell lung carcinoma) OR (non-small cell lung carcinoma) OR (non small cell lung cancer) OR (non-small cell lung cancer) OR (pulmonary carcinomas) OR (pulmonary carcinoma) OR (pulmonary neoplasms) OR (lung carcinomas) OR (lung malignan*) OR (Lung malignan*) OR (lung malignancies) OR (pulmonary neoplasm) OR (lung carcinoma) OR (lung malignancy) OR (lung neoplasms) OR (lung neoplasm) OR (lung cancer) OR ("Lung Neoplasms"[Mesh])
Hits: 431,599.

Citation: Wen, S.W.C.; Wen, J.; Hansen, T.F.; Jakobsen, A.; Hilberg, O. Cell Free Methylated Tumor DNA in Bronchial Lavage as an Additional Tool for Diagnosing Lung Cancer—A Systematic Review. *Cancers* **2022**, *14*, 2254. https://doi.org/10.3390/cancers14092254

Academic Editors: Danijela Koppers-Lalic, Vesselin Baev and Bruno Costa Silva

Received: 31 March 2022
Accepted: 28 April 2022
Published: 30 April 2022

Publisher's Note: MDPI stays neutral with regard to jurisdictional claims in published maps and institutional affiliations.

Copyright: © 2022 by the authors. Licensee MDPI, Basel, Switzerland. This article is an open access article distributed under the terms and conditions of the Creative Commons Attribution (CC BY) license (https://creativecommons.org/licenses/by/4.0/).

Block 2:
(bronchoalveolar lavage[MeSH Terms]) OR (bronchoalveolar lavage fluid[MeSH Terms]) OR (bronchial lavage) OR (bronchial lavages) OR (bronchial lavage*) OR (bronchial lavage fluid) OR (bronchial lavage fluids) OR (bronchial lavage fluid*) OR (bronchoalveolar lavage) OR (bronchoalveolar lavages) OR (bronchoalveolar lavage*) OR (bronchoalveolar lavage fluid) OR (bronchoalveolar lavage fluids) OR (bronchoalveolar lavage fluid*) OR (bronchioalveolar lavage) OR (bronchioalveolar lavages) OR (bronchioalveolar lavage*) OR (bronchioalveolar lavage fluid) OR (bronchioalveolar lavage fluids) OR (bronchial wash) OR (bronchial washes) OR (bronchial washing) OR (bronchial washings) OR (bronchial wash*) OR (bronchial fluid) OR (bronchial fluids) OR (bronchial fluid*) OR (lung lavage) OR (lung lavages) OR (lung lavage*) OR (bronchopulmonary lavage) OR (Bronchopulmonary lavages) OR (Bronchopulmonary lavage*) OR (Pulmonary lavage fluid) OR (Pulmonary lavage fluids) OR (Pulmonary lavage

fluid*) OR (Bronchoalveolar wash) OR (Bronchoalveolar washes) OR (Bronchoalveolar washing) OR (Bronchoalveolar washings) OR (Bronchoalveolar wash*) OR (Bronchioalveolar wash) OR (Bronchioalveolar washes) OR (Bronchioalveolar washing) OR (Bronchioalveolar washings) OR (Bronchioalveolar wash*) OR (Bronchopulmonary wash) OR (Bronchopulmonary washes) OR (Bronchopulmonary washing) OR (Bronchopulmonary washings) OR (Bronchopulmonary wash*) OR (Pulmonary wash) OR (Pulmonary washes) OR (Pulmonary washing) OR (Pulmonary washings) OR (Pulmonary wash*) OR (Lung wash) OR (Lung washes) OR (Lung washing) OR (Lung washings) OR (Lung wash*)
Hits: 80,825.

Block 3:
(liquid biops*) OR (liquid biopsies) OR (liquid biopsy) OR (liquid biopsy[MeSH Terms]) OR (Genetic methylat*) OR (Genetic methylations) OR (Genetic methylation) OR (methylat*) OR (methylations) OR (methylated) OR (methylation) OR (methylat* gene) OR (methylated gene) OR (Gene methylat*) OR (gene methylations) OR (gene methylation) OR (methylat* dna) OR (DNA methylat*) OR (DNA methylations) OR (dna methylation) OR (methylated dna) OR (cfdna) OR (ctdna) OR (circulating dna) OR (Circulating tumor-specific DNA) OR (Circulating tumor specific DNA) OR (Circulating cell-free tumor DNA) OR (Circulating cell free tumor DNA) OR (circulating tumor dna) OR (cell-free tumor dna) OR (cell free tumor dna) OR (cell-free dna) OR (cell free dna) OR (tumor dna) OR (DNA, neoplasm[MeSH Terms]) OR (circulating tumor dna[MeSH Terms])
Hits: 865,994.

Block 1 AND block 2 AND block 3
Hits: 769.
Filter, English. Hits: 746.

Embase Classic + Embase from 1947: Searched on February 25, 2022.

Block 1:
exp lung cancer/ OR lung cancer/ OR lung cancer.mp. OR (lung adj3 cancer).mp. OR lung neoplasm.mp. OR lung neoplasms.mp. OR (lung adj3 neoplasm).mp. OR (lung adj3 neoplasms).mp. OR lung malignancy.mp. OR (lung adj3 malignancy).mp. OR lung malignancies.mp. OR (lung adj3 malignancies).mp. OR lung maligna*.mp. OR (lung adj3 maligna*).mp. OR lung carcinoma.mp. OR (lung adj3 carcinoma).mp. OR lung carcinomas.mp. OR (lung adj3 carcinomas).mp. OR pulmonary neoplasm.mp. OR (pulmonary adj3 neoplasm).mp. OR pulmonary neoplasms.mp. OR (pulmonary adj3 neoplasms).mp. OR pulmonary carcinoma.mp. OR (pulmonary adj3 carcinoma).mp. OR pulmonary carcinomas.mp. OR (pulmonary adj3 carcinomas).mp. OR pulmonary cancer.mp. OR (pulmonary adj3 cancer).mp. OR pulmonary cancers.mp. OR (pulmonary adj3 cancers).mp. OR non-small cell lung cancer.mp. OR exp lung non small cell cancer/ OR exp non small cell lung cancer/ OR non small cell lung cancer.mp. OR non-small cell lung carcinoma.mp. OR non small cell lung carcinoma.mp. OR non-small cell lung carcinomas.mp. OR non small cell lung carcinomas.mp. OR small cell lung cancer.mp. OR small cell lung cancers.mp. OR small cell lung carcinoma.mp. OR small cell lung carcinomas.mp. OR (small cell adj3 lung adj3 cancer).mp. OR (small cell adj3 lung adj3 carcinoma).mp. OR (non small cell adj3 lung adj3 cancer).mp. OR (non-small cell adj3 lung

adj3 cancer).mp. OR (non small cell adj3 lung adj3 carcinoma).mp. OR (non-small cell adj3 lung adj3 carcinoma).mp. OR adenocarcinoma of the lung.mp. OR lung adenocarcinoma.mp. OR (lung adj3 adenocarcinoma).mp. OR (lung adj3 adenocarcinomas).mp. OR squamous cell carcinoma of the lung.mp. OR lung squamous cell carcinoma.mp. OR (lung adj3 squamous cell carcinoma).mp. OR squamous cell lung cancer.mp. OR (squamous cell adj3 lung adj3 cancer).mp. OR bronchial carcinoma.mp. OR bronchial carcinomas.mp. OR (bronchial adj3 carcinoma).mp. OR (bronchial adj3 carcinomas).mp. OR bronchial neoplasm.mp. OR bronchial neoplasms.mp. OR (bronchial adj3 neoplasm).mp. OR (bronchial adj3 neoplasms).mp. OR carcinoma bronchogenic.mp. OR (carcinoma adj3 bronchogenic).mp. OR Bronchiolo alveolar adenocarcinoma.mp. OR Bronchiolo alveolar adenocarcinomas.mp. OR Bronchiolo-alveolar adenocarcinoma.mp. OR Bronchiolo-alveolar adenocarcinoma.mp. OR Bronchiolo-alveolar adenocarcinomas.mp. OR (Bronchiolo adj3 alveolar adj3 adenocarcinoma).mp. OR Schneeberg disease.mp. OR (Schneeberg adj3 disease).mp.
Hits: 514,575.

Block 2:
exp lung lavage/ OR exp bronchoalveolar lavage fluid/ OR bronchial lavage.mp. OR bronchial lavages.mp. OR (bronchial adj3 lavage).mp. OR (bronchial adj3 lavage*).mp. OR bronchial lavage fluid.mp. OR bronchial lavage fluids.mp. OR (bronchial adj3 lavage adj3 fluid).mp. OR Bronchoalveolar lavage.mp. OR (Bronchoalveolar adj3 lavage).mp. OR (Bronchoalveolar adj3 lavages).mp. OR Bronchoalveolar lavages.mp. OR Bronchoalveolar lavage*.mp. OR (Bronchoalveolar adj3 lavage*).mp. OR Bronchoalveolar lavage fluid.mp. OR Bronchoalveolar lavage fluids.mp. OR (Bronchoalveolar adj3 lavage adj3 fluid).mp. OR Bronchoalveolar lavage fluid*.mp. OR Bronchioalveolar lavage.mp. OR (Bronchioalveolar adj3 lavage).mp. OR Bronchioalveolar lavages.mp. OR (Bronchioalveolar adj3 lavages).mp. OR (Bronchioalveolar adj3 lavage*).mp. OR Bronchioalveolar lavage fluid.mp. OR Bronchioalveolar lavage fluids.mp. OR (Bronchioalveolar adj3 lavage adj3 fluid).mp. OR (Bronchioalveolar adj3 lavage adj3 fluid*).mp. OR Bronchial wash.mp. OR Bronchial washes.mp. OR Bronchial washing.mp. OR Bronchial washings.mp. OR (Bronchial adj3 wash*).mp. OR Bronchial fluid.mp. OR Bronchial fluids.mp. OR (Bronchial adj3 fluid).mp. OR (Bronchial adj3 fluids).mp. OR (Bronchial adj3 fluid*).mp. OR lung lavage.mp. OR Lung lavages.mp. OR (Lung adj3 lavage).mp. OR (Lung adj3 lavages).mp. OR (Lung adj3 lavage*).mp. OR Bronchopulmonary lavage.mp. OR Bronchopulmonary lavages.mp. OR Bronchopulmonary lavag*.mp. OR (Bronchopulmonary adj3 lavage).mp. OR (Bronchopulmonary adj3 lavag*).mp. OR Pulmonary lavage fluid.mp. OR Pulmonary lavage fluids.mp. OR Pulmonary lavage fluid*.mp. OR Bronchoalveolar wash.mp. OR Bronchoalveolar washes.mp. OR Bronchoalveolar washing.mp. OR Bronchoalveolar washings.mp. OR Bronchoalveolar wash*.mp. OR Bronchioalveolar wash.mp. OR Bronchioalveolar washes.mp. OR Bronchioalveolar washing.mp. OR Bronchioalveolar washings.mp. OR Bronchioalveolar wash*.mp. OR Bronchopulmonary wash.mp. OR Bronchopulmonary washes.mp. OR Bronchopulmonary washing.mp. OR Bronchopulmonary washings.mp. OR Bronchopulmonary wash*.mp. OR Pulmonary wash.mp. OR Pulmonary washes.mp. OR Pulmonary washings.mp. OR Pulmonary washings.mp. OR Pulmonary wash*.mp. OR Lung wash.mp. OR Lung washes.mp. OR Lung washings.mp. OR Lung washing.mp. OR Lung wash*.mp.
Hits: 78,074.

Block 3:

exp circulating tumor DNA/ OR exp liquid biopsy/ OR tumor dna.mp. OR tumour dna.mp OR (tumor adj3 dna).mp. OR (tumour adj3 dna).mp. OR cell free dna.mp. OR cell-free dna.mp. OR cell free tumor dna.mp. OR (cell free adj3 tumor adj3 dna).mp. OR (cell free adj3 tumour adj3 dna).mp. OR circulating tumor dna.mp. OR (circulating adj3 tumor adj3 dna).mp. OR circulating tumour dna.mp. OR (circulating adj3 tumour adj3 dna).mp. OR Circulating cell free tumor DNA.mp. OR (Circulating adj3 cell free adj3 tumor DNA).mp. OR (Circulating adj3 cell free adj3 tumour DNA).mp. OR Circulating cell free tumour DNA.mp. OR Circulating tumor specific DNA.mp. OR Circulating tumour specific DNA.mp. OR (Circulating adj3 tumor specific DNA).mp. OR (Circulating adj3 tumour specific DNA).mp. OR Circulating DNA.mp. OR ctdna.mp. OR cfdna.mp. OR Methylated DNA.mp. OR (Methylated adj3 DNA).mp. OR DNA methylation.mp. OR DNA methylations.mp. OR DNA methylation*.mp. OR DNA methylat*.mp. OR (DNA adj3 methylat*).mp. OR Gene methylation.mp. OR Gene methylations.mp. OR Gene methylat*.mp. OR (Gene adj3 methylation).mp. OR Gene* methylation.mp. OR (Gene adj3 methylat*).mp. OR (Gene* adj3 methylat*).mp. OR methylated gene.mp. OR methylat* gene.mp. OR methylation.mp. OR methylations.mp. OR methylated.mp. OR methylat*.mp. OR genetic methylation.mp. OR genetic methylations.mp. OR (genetic adj3 methylat*).mp. OR liquid biopsy.mp. OR liquid biopsies.mp. OR liquid biops*.mp.
Hits: 241,736.

Block 1 AND block 2 AND block 3
Hits: 173 hits.

Medline: Searched on March 9, 2022.

Same search strings as Embase.
Block 1 AND block 2 AND block 3
Hits: 87.

Web of Science Core Collection: Searched March 1, 2022.

Block 1:
TS=(lung cancer) OR TS=(lung neoplasm) OR TS=(lung malignancy) OR TS=(lung malignan*) OR TS=(lung neoplas*) OR TS=(lung carcinoma) OR TS=(lung carcino*) OR TS=(Pulmonary neoplasm) OR TS=(Pulmonary neoplas*) OR TS=(Pulmonary carcinoma) OR TS=(Pulmonary carcino*) OR TS=(Pulmonary cancer) OR TS=(Pulmonary cancer*) OR TS=(Non-small cell lung cancer) OR TS=(Non small cell lung cancer) OR TS=(Non-small cell lung carcinoma) OR TS=(Non small cell lung carcinoma) OR TS=(Small cell lung cancer) OR TS=(Small cell lung carcinoma) OR TS=(Adenocarcinoma of the lung) OR TS=(Lung adenocarcinoma) TS=(Squamous cell carcinoma of the lung) OR TS=(Lung squamous cell carcinoma) OR TS=(pulmonary squamous cell carcinoma) OR TS=(pulmonary adenocarcinoma) OR TS=(Squamous cell lung cancer) OR TS=(Bronchial carcinoma) OR TS=(Bronchial neoplasms) OR TS=(Carcinoma, bronchogenic) OR TS=(Bronchiolo alveolar adenocarcinoma) OR TS=(Schneeberg disease)
Hits: 448,795.

Block 2:

TS=(pulmonary lavage) OR TS=(bronchoalveolar wash*) OR TS=(bronchioalveolar wash*) OR TS=(bronchopulmonary wash*) OR TS=(pulmonary wash*) OR TS=(bronchial wash*) OR TS=(lung wash*) OR TS=(lung washing) OR TS=(lung wash) OR TS=(bronchial washing) OR TS=(Pulmonary washing) OR TS=(Pulmonary wash) OR TS=(Bronchopulmonary washing) OR TS=(Bronchopulmonary wash) OR TS=(Bronchioalveolar washing) OR TS=(Bronchioalveolar wash) OR TS=(Bronchoalveolar washing) OR TS=(Bronchoalveolar wash) OR TS=(Pulmonary lavage fluid) OR TS=(Bronchopulmonary lavage) OR TS=(lung lavage) OR TS=(bronchial fluid) OR TS=(Bronchial wash fluid) OR TS=(Bronchial wash) OR TS=(Bronchioalveolar lavage fluid) OR TS=(Bronchioalveolar lavage) OR TS=(Bronchoalveolar lavage fluid) OR TS=(bronchoalveolar lavage) OR TS=(bronchial lavage fluid) OR TS=(bronchial lavage)
Hits: 51,807.

Block 3:
TS=(liquid biops*) OR TS=(liquid biopsies) OR TS=(liquid biopsy) OR TS=(genetic methylat*) OR TS=(genetic methylations) OR TS=(genetic methylation) OR TS=(methylat*) OR TS=(methylated) OR TS=(methylations) OR TS=(methylation) OR TS=(methylated gene) OR TS=(gene methylat*) OR TS=(gene methylations) OR TS=(gene methylation) OR TS=(dna methylat*) OR TS=(dna methylations) OR TS=(dna methylation) OR TS=(methylated dna) OR TS=(cfdna) OR TS=(ctdna) OR TS=(circulating tumor-specific dna) OR TS=(circulating tumor specific dna) OR TS=(circulating cell free tumour dna) OR TS=(circulating cell-free tumor dna) OR TS=(circulating cell free tumor dna) OR TS=(circulating tumour dna) OR TS=(circulating tumor dna) OR TS=(cell-free tumour dna) OR TS=(cell free tumour dna) OR TS=(cell free tumor dna) OR TS=(cell-free tumor dna) OR TS=(cell-free dna) OR TS=(cell free dna) OR TS=(tumour dna) OR TS=(tumor dna)
Hits: 386,411.

Block 1 AND block 2 AND block 3
Hits: 349.

2. Detailed quality assessments of the included studies

Table S1: Patient selection

Study ID	1. Patient selection. Was a consecutive or random sample of patients enrolled?	Was a case-control design avoided?	Did the study avoid inappropriate exclusions?	Could the selection of patients have introduced bias?	Is there concern that the included patients do not match the review question?
Kersting 2000 [22]	Yes	No	Unclear	Low risk	Low risk
Kim 2004 [23]	Unclear	No	Yes	Low risk	Low risk
Topaloglu 2004 [24]	Unclear	No	Unclear	Unclear risk	Low risk
de Fraipont 2005 [25]	Yes	No	Unclear	Unclear risk	Low risk

Grote 2005 [26]	Yes	No	Yes	Low risk	Low risk
Schmiemann 2005 [27]	Yes	No	Yes	Low risk	Low risk
Schmidt 2010 [28]	No	No	Unclear	Unclear risk	Low risk
Schramm 2011 [9]	Yes	No	Yes	Low risk	Low risk
Dietrich 2012 [29]	Unclear	No	Unclear	High risk	Low risk
Nikolaidis 2012 [30]	No	No	Yes	High risk	Low risk
van der Drift 2012 [10]	Unclear	No	Unclear	Unclear risk	Low risk
Diaz-Lagares 2016 [31]	No	No	Unclear	High risk	Low risk
Konecny 2016 [32]	Unclear	No	Unclear	Unclear risk	Low risk
Ren 2017 [33]	Unclear	No	Unclear	Unclear risk	Low risk
Zhang 2017 [34]	Unclear	No	Yes	High risk	Low risk
Feng 2018 [35]	Unclear	No	Unclear	Unclear risk	Low risk
Jeong 2018 [36]	Unclear	No	Yes	Low risk	Low risk
Um 2018 [37]	No	No	Yes	High risk	Low risk
Dong 2019 [38]	Unclear	No	Unclear	Unclear risk	Low risk
Villalba 2019 [39]	No	No	Unclear	Unclear risk	Low risk
Rizk 2020 [40]	Unclear	No	Unclear	Unclear risk	Low risk
Roncarati 2020 [41]	Yes	No	Yes	Low risk	Low risk
Li 2021 [42]	Unclear	No	Unclear	Unclear risk	Low risk
Wen 2021 [43]	Yes	No	Yes	Low risk	Low risk
Zeng 2021 [44]	Yes	No	Yes	Low risk	Low risk

Table S2: Index test

**Study ID**	**2. Index test. Were the test results interpreted without knowledge of the results of the reference standard?**	**If a threshold was used, was it pre-specified?**	**Could the conduct or interpretation of the index test have introduced bias?**	**Is there concern that the index test, its conduct or interpretation differ from the review question?**
Kersting 2000 [22]	Unclear	Unclear	Unclear risk	Low risk
Kim 2004 [23]	Unclear	Unclear	Unclear risk	Low risk
Topaloglu 2004 [24]	Unclear	No	High risk	Low risk
de Fraipont 2005 [25]	Unclear	Unclear	Unclear risk	Low risk
Grote 2005 [26]	Yes	No	Low risk	Low risk

Schmiemann 2005 [27]	Yes	Yes	Low risk	Low risk
Schmidt 2010 [28]	Unclear	Unclear	Unclear risk	Low risk
Schramm 2011 [9]	Yes	Yes	Low risk	Low risk
Dietrich 2012 [29]	Unclear	Yes	High risk	Low risk
Nikolaidis 2012 [30]	Unclear	Yes	Unclear risk	Low risk
van der Drift 2012 [10]	Unclear	Unclear	Unclear risk	Low risk
Diaz-Lagares 2016 [31]	Unclear	Yes	Unclear risk	Low risk
Konecny 2016 [32]	Unclear	Unclear	Unclear risk	Low risk
Ren 2017 [33]	Unclear	Unclear	Unclear risk	Low risk
Zhang 2017 [34]	Unclear	Unclear	High risk	Low risk
Feng 2018 [35]	Unclear	Unclear	Unclear risk	Low risk
Jeong 2018 [36]	Unclear	No	Unclear risk	Low risk
Um 2018 [37]	Unclear	Yes	Low risk	Low risk
Dong 2019 [38]	Unclear	Yes	Unclear risk	Low risk
Villalba 2019 [39]	Unclear	No	High risk	Low risk
Rizk 2020 [40]	Unclear	No	Unclear risk	Low risk
Roncarati 2020 [41]	Unclear	No	Unclear risk	Low risk
Li 2021 [42]	Unclear	Yes	Unclear risk	Low risk
Wen 2021 [43]	Yes	Yes	Low risk	Low risk
Zeng 2021 [44]	Unclear	Yes	Low risk	Low risk

Table S3: Reference standard

**Study ID**	**3. Reference standard. Is the reference standard likely to correctly classify the target condition?**	**Were the reference standard results interpreted without knowledge of the index test results?**	**Could the reference standard, its conduct, or its interpretation have introduced bias?**	**Is there concern that the target condition as defined by the reference standard does not match the review question?**
Kersting 2000 [22]	Yes	Yes	Low risk	Low risk

Kim 2004 [23]	Yes	Unclear	Low risk	Low risk
Topaloglu 2004 [24]	Yes	Yes	Low risk	Low risk
de Fraipont 2005 [25]	Yes	Unclear	Unclear risk	Low risk
Grote 2005 [26]	Yes	Yes	Low risk	Low risk
Schmiemann 2005 [27]	Yes	Yes	Low risk	Low risk
Schmidt 2010 [28]	Yes	Yes	Low risk	Low risk
Schramm 2011 [9]	Yes	Yes	Low risk	Low risk
Dietrich 2012 [29]	Yes	Yes	Low risk	Low risk
Nikolaidis 2012 [30]	Yes	Unclear	Low risk	Low risk
van der Drift 2012 [10]	Yes	Unclear	Unclear risk	Low risk
Diaz-Lagares 2016 [31]	Yes	Unclear	Low risk	Low risk
Konecny 2016 [32]	Yes	Unclear	Unclear risk	Low risk
Ren 2017 [33]	Yes	Unclear	Low risk	Low risk
Zhang 2017 [34]	Yes	Unclear	Low risk	Low risk
Feng 2018 [35]	Yes	Unclear	Unclear risk	Low risk
Jeong 2018 [36]	Yes	Unclear	Unclear risk	Low risk
Um 2018 [37]	Yes	Unclear	Low risk	Low risk
Dong 2019 [38]	Unclear	Unclear	Unclear risk	Low risk
Villalba 2019 [39]	Yes	Yes	Low risk	Low risk
Rizk 2020 [40]	Yes	Unclear	Unclear risk	Low risk
Roncarati 2020 [41]	Yes	Unclear	Low risk	Low risk
Li 2021 [42]	Yes	Unclear	Low risk	Low risk
Wen 2021 [43]	Yes	Yes	Low risk	Low risk
Zeng 2021 [44]	Yes	Yes	Low risk	Low risk

Table S4: Flow and timing

**Study ID**	**4. Flow and timing. Was there an appropriate interval between index test**	**Did all patients receive a reference standard?**	**Did patients receive the same reference standard?**	**Were all patients included in the analysis?**	**Could the patient flow have introduced bias?**

	**and reference standard?**				
Kersting 2000 [22]	Unclear	Yes	Yes	Yes	Low risk
Kim 2004 [23]	Yes	Yes	Yes	Yes	Low risk
Topaloglu 2004 [24]	Yes	Yes	Yes	Yes	Low risk
de Fraipont 2005 [25]	Yes	Yes	No	Yes	Low risk
Grote 2005 [26]	Yes	Yes	No	No	Low risk
Schmiemann 2005 [27]	Yes	Yes	No	Yes	Low risk
Schmidt 2010 [28]	Yes	Yes	No	Yes	Low risk
Schramm 2011 [9]	Yes	Yes	No	Yes	Low risk
Dietrich 2012 [29]	Yes	Yes	No	No	Low risk
Nikolaidis 2012 [30]	Yes	Yes	Unclear	Yes	Low risk
van der Drift 2012 [10]	Yes	Yes	No	Yes	Low risk
Diaz-Lagares 2016 [31]	Yes	Yes	Unclear	Yes	Low risk
Konecny 2016 [32]	Yes	Yes	No	Yes	Low risk
Ren 2017 [33]	Yes	Yes	Yes	Yes	Low risk
Zhang 2017 [34]	Yes	Yes	Yes	Yes	Low risk
Feng 2018 [35]	Yes	Yes	Yes	Yes	Low risk
Jeong 2018 [36]	Yes	Yes	No	No	Low risk
Um 2018 [37]	Yes	Yes	Yes	Yes	Low risk
Dong 2019 [38]	Unclear	Unclear	Unclear	Yes	Unclear
Villalba 2019 [39]	Yes	Yes	Yes	No	Low risk
Rizk 2020 [40]	Unclear	Yes	No	Yes	Low risk
Roncarati 2020 [41]	Yes	Yes	Yes	No	Low risk
Li 2021 [42]	Yes	Yes	Yes	No	Low risk
Wen 2021 [43]	Yes	Yes	No	Yes	Low risk
Zeng 2021 [44]	Yes	Yes	Yes	Yes	Low risk

Table S5: Conflicts of interest

**Study ID**	**Did the authors report any conflicts of interest?**	**Did the authors report any conflicts of interest? supporting text**
Kersting 2000 [22]	Unclear	
Kim 2004 [23]	No	
Topaloglu 2004 [24]	Yes	Oncogenome Sciences.
de Fraipont 2005 [25]	Unclear	
Grote 2005 [26]	Unclear	
Schmiemann 2005 [27]	Unclear	
Schmidt 2010 [28]	Yes	Volker Liebenberg, Dimo Dietrich, Thomas Schlegel, Christoph Kneip, Anke Seegebarth, Nadja Flemming, Stefanie Seemann, Jörn Lewin, Juergen Distler, Ulrike Wille, and Reimo Tetzner are or have been employees and/or stockholders of Epigenomics AG, a company that aims to commercialize DNA methylation markers. John Field is a member of the EpigenomicsAdvisory Board.
Schramm 2011 [9]	Yes	Motic Company, China
Dietrich 2012 [29]	No	But they performed the study in order to get a CE marking for the Epi proLung BL Reflex Assay.
Nikolaidis 2012 [30]	Yes	J.R. Gosney has honoraria from Speakers Bureau and is a consultant/advisoryboard member of Eli Lilly, AstraZeneca, and Pfizer. No potential conflicts ofinterest were disclosed by the other authors.
van der Drift 2012 [10]	No	
Diaz-Lagares 2016 [31]	Yes	J . Zulueta has ownership interest (including patents) in VisionGate, Inc. Nopotential conflicts of interest were disclosed by the other authors
Konecny 2016 [32]	Unclear	
Ren 2017 [33]	No	
Zhang 2017 [34]	No	
Feng 2018 [35]	No	
Jeong 2018 [36]	No	
Um 2018 [37]	No	
Dong 2019 [38]	Yes	W. Yu and Shihua Dong report having a pending patent application.
Villalba 2019 [39]	No	
Rizk 2020 [40]	No	

Roncarati 2020 [41]	No	
Li 2021 [42]	Yes	Zhujia Ye, Hao Yang, Jinsheng Tao, Siyu Chen, Jiehan Xu, Yanying Liu, Weihe Liang, Bo Wang, Zhiwei Chen, Jian-Bing Fan are employees of AnchorDx Medi-cal Co, Ltd. or AnchorDx, Inc. All other authors declare no competing financial interest.
Wen 2021 [43]	Yes	NK Max outside of the submitted work.
Zeng 2021 [44]	Yes	Many authors were employed by Burning Rock Biotech.

3. Diagnostic properties of all genes in the included studies

Table S6: Sensitivity and specificity of included studies

**Study ID**	**Gene name**	**Sens**	**Spec**	**AUC**
2000 Kersting [22]	p16INK4a	22%	88%	
2004 Kim [23]	p16	16.5%	93.7%	
	RASSF1A	17.6%	96.1%	
	FHIT	22.4%	71.7%	
	H-cadherin	12.9%	96.9%	
	RAR-b	15.3%	87.4%	
2004 Topaloglu [24]	CDH1	48 %	100 %	
	APC	29 %	100 %	
	MGMT	58 %	100 %	
	RASSF1A	29 %	100 %	
	GSTP1	33 %	100 %	
	p16	14%		
	RAR-b2	0%		
	ARF			
2005 de Fraipont [25]	p16	15.2%	81.4%	
	DAPK	5.9%	95.3%	
	MGMT	20.6%	79.1%	
	FHIT	29.4%	66.7%	
	APC	0 %	93.1%	
	p16, cancer	11.8%		
	DAPK, cancer	16.7%		
	MGMT, cancer	5.6%		
	FHIT, cancer	37.5%		
	APC, cancer	0 %		
	At least one gene: sens 55.9%, spec 44.2% for all. Excluding preinvasive lesions sens 55.6%, spec 44.2%.			

2005 Grote [26]	p16INK4a	24 %	100 %	
	RARB2	56% calculated (they report 80%)	87.5% calculated (they report 52%)	
	SEMA3B	88 %	8 %	
2005 Schmiemann [27]	APC	16.5%	99 %	
	p16INK4a	11.8%	100 %	
	RAR-b2	47.1%	79.4%	
	RASSF1A	41.2%	100 %	
	APC, p16, RASSF1A	53 %	99 %	
	APC, p16, RASSF1A, RARB2	71 %	78 %	
2010 Schmidt [28]	SHOX2	68 %	95 %	86 %
2011 Schramm [9]	APC, p16INK4A, RASSF1A, whole cohort.	49.6%	98.4%	
	APC, p16INK4A, RASSF1A, Equivocal cases	49.0%	95 %	
	RASSF1A			
2012 Dietrich [29]	SHOX2	78 %	96 %	94 %
2012 Nikolaidis [30]	p16, TERT, WT1, and RASSF1			
	TERT test / validation	67.0% / 54.0%	83.6% / 98.2%	
	RASSF1 test / validation	38.7% / 51.1%	96.7% / 100%	
	WT1 test / validation	36.1% / 52.5%	95.3% / 93.5%	
	p16 test / validation	18.6% / 12.9%	99.5% / 100%	
	CYGB test / validation	18.6% / 10.8%	92.5% / 100%	
	RARbeta test / validation	14.4% / 48.2%	95.3% / 83.5%	
	Combined 6 test / validation	79.4% / 82.0%	79.8% / 90.8%	
	4 genes and demographic data validation	82 %	91 %	
2012 van der Drift [10]	RASSF1A	42.6%	96.4%	
2016 Diaz-Lagares [31]	Combined model, aspirates (BCAT1, CDO1, TRIM58, ZNF177)	84.6%	81 %	90 %

				0.91 [95% CI (0.83–0.98) *p* < 0.001
	BCAT1, BAL			80 %
	CDO1, BAL			65 %
	TRIM58, BAL			72 %
	ZNF177, BAL			66 %
	Combined model, BAL			83 % 95% CI (0.78–0.93) *p* < 0.001
2016 Konecny [32]	SHOX2	83.8%	84.6%	
2017 Ren [33]	SHOX2	64.2%	92.9%	
	RASSF1A	50.4%	95.5%	
	Any of the two	71.5%	90.2%	
2017 Zhang [34]	SHOX2			
	SHOX2, RASSF1A (combined model)	81.0%	97.4%	
2018 Feng [35]	NID2	30.4%	83 %	63.2%
2018 Jeong [36]	PCDHGA12	75 %	78.9%	81.9%
2018 Um [37]	TFAP2A, TBX15, PRR15, HOXA11, PDGFRA, TOX2, PHF11 combined in a logit model	87.0%	83.3%	87 %
2019 Dong [38]	Combined marker, training	78.6%	96.7%	0.91; 95% CI, 0.86–0.96; *p*<0.0001
	Combined marker, validation	82.5%	96.5%	
2019 Villalba [39]	TMPRSS4			59% (95% CI 47-71%)
	SHOX2			71% (95% CI 60-81%)
	TMPRSS4 early stage	52 %	91 %	
	SHOX2 early stage	86 %	60 %	
	Combined			76% (95% CI 62-86%)
2020 Rizk [40]	SHOX2	77 %	70 %	91 %
2020 Roncarati [41]	RASSF1A	46 %	100 %	
	CDH1	64 %	74 %	
	DLC1	37 %	94 %	
	PRPH	40 %	100 %	
	Any methylation positive	97 %	74 %	93 %

2021 Li [42]	PTGER4-1	73	60	
	PTGER4-2	75	73	
	HOXB4-1	74	71	
	HOXB4-2	67	64	
	HOXB4-3	71	73	
	HOXB4-4	48	81	
	IHX9	59	77	
	GSHR	62	62	
	MIR196A1	58	78	
	HOXA11	55	79	
	CDO1	55	84	72
	5 gene model, test	82	91	
	5 gene model, validation	70	82	
2021 Wen [43]	HOXA9, test	73.1%	85.3%	
	HOXA9, validation	80.0%	75.6%	
2021 Zeng [44]	Methylation model	81 %	81 %	

Figure S1: Forest plot of sensitivity and specificity for the SHOX2 gene

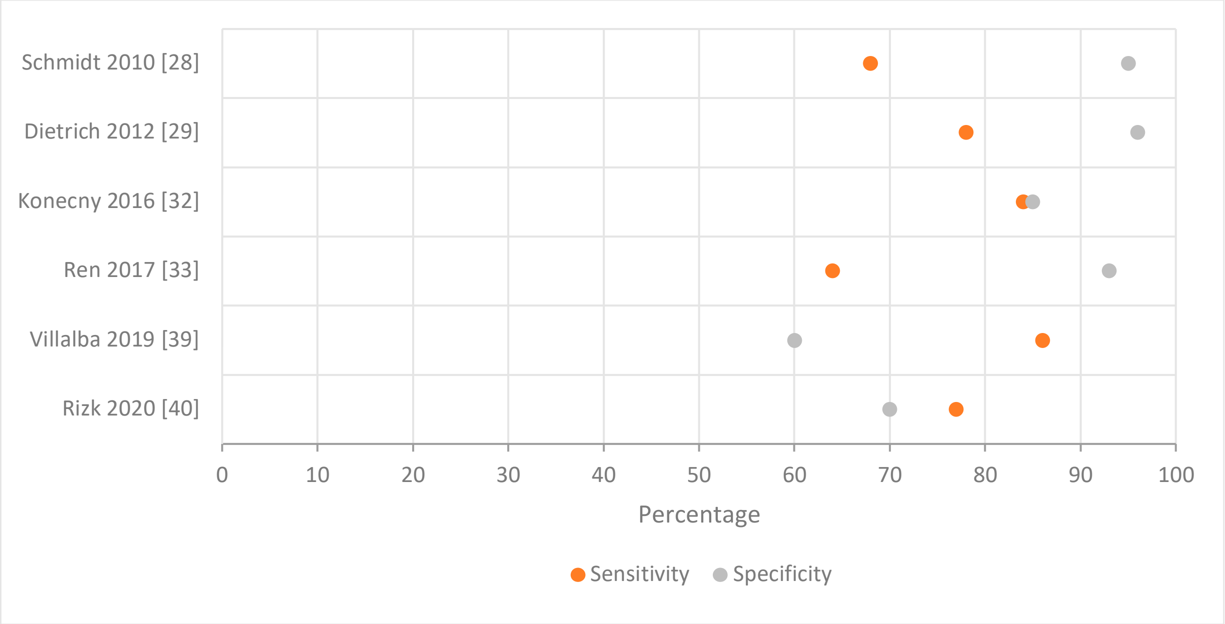

Figure S1. Forest plot illustrating the sensitivity (orange) and specificity (gray) of the SHOX2 gene. The x-axis represents study sensitivity and specificity in percent. There are no whiskers, since many studies did not report a 95% confidence interval.

Figure S2: Forest plot of sensitivity and specificity for the RARbeta2 gene

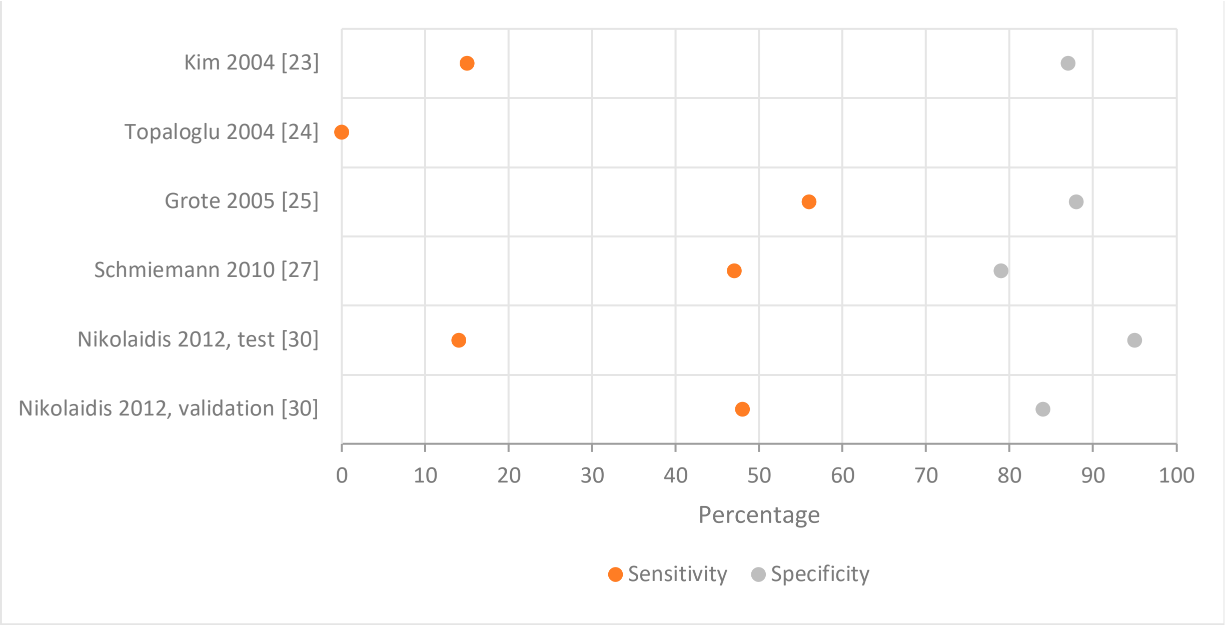

Figure S2. Forest plot illustrating the sensitivity (orange) and specificity (gray) of the RARbeta2 gene. If a test and validation approach was used, both cohorts were included in the graph. The x-axis represents study sensitivity and specificity in percent. There are no whiskers, since many studies did not report a 95% confidence interval.
